# The current state of intensive care unit discharge practices - Results of an international survey study

**DOI:** 10.3389/fmed.2024.1377902

**Published:** 2024-05-07

**Authors:** Maike Hiller, Christian Burisch, Maria Wittmann, Hendrik Bracht, Arnold Kaltwasser, Jan Bakker

**Affiliations:** ^1^Department of Intensive Care Adults, Erasmus MC University Medical Center, Rotterdam, Netherlands; ^2^Department of Hospital Patient Monitoring, Philips Medizin Systeme Böblingen GmbH, Böblingen, Germany; ^3^Regional Government Düsseldorf, State of North Rhine-Westphalia, Düsseldorf, Germany; ^4^Department of Anesthesiology and Intensive Care Medicine, University Hospital Bonn, Bonn, Germany; ^5^Department of Anesthesiology, Intensive Care, Emergency and Transfusion Medicine and Pain Therapy, University Hospital Bielefeld Bethel, Campus Bielefeld-Bethel, Bielefeld, Germany; ^6^Academy of the District Hospitals Reutlingen, Kreiskliniken Reutlingen, Reutlingen, Germany; ^7^New York University School of Medicine and Columbia University College of Physicians and Surgeons, New York, NY, United States; ^8^Department of Intensive Care, Pontifcia Universidad Catolica de Chile, Santiago de Chile, Chile

**Keywords:** survey, intensive care unit discharge, discharge process, discharge criteria, discharge tools, discharge barriers, care transitions, patient transfer

## Abstract

**Background:**

Increasing pressure on limited intensive care capacities often requires a subjective assessment of a patient's discharge readiness in the absence of established Admission, Discharge, and Transfer (ADT) guidelines. To avoid suboptimal care transitions, it is important to define clear guidelines for the admission and discharge of intensive care patients and to optimize transfer processes between the intensive care unit (ICU) and lower care levels. To achieve these goals, structured insights into usual ICU discharge and transfer practices are essential. This study aimed to generate these insights by focusing on involved stakeholders, established processes, discharge criteria and tools, relevant performance metrics, and current barriers to a timely and safe discharge.

**Method:**

In 2022, a structured, web-based, anonymous cross-sectional survey was conducted, aimed at practicing ICU physicians, nurses, and bed coordinators. The survey consisted of 29 questions (open, closed, multiple choice, and scales) that were divided into thematic blocks. The study was supported by several national and international societies for intensive care medicine and nursing.

**Results:**

A total of 219 participants from 40 countries (105 from Germany) participated in the survey. An overload of acute care resources with ~90% capacity utilization in the ICU and the general ward (GW) leads to not only premature but also delayed patient transfers due to a lack of available ward and intermediate care (IMC) beds. After multidisciplinary rounds within the intensive care team, the ICU clinician on duty usually makes the final transfer decision, while one-third of the panel coordinates discharge decisions across departmental boundaries. By the end of the COVID-19 pandemic, half of the hospitals had implemented ADT policies. Among these hospitals, nearly one-third of the hospitals had specific transfer criteria established, consisting primarily of vital signs and laboratory data, patient status and autonomy, and organization-specific criteria. Liaison nurses were less common but were ranked right after the required IMC capacities to bridge the care gap between the ICU and normal wards. In this study, 80% of the participants suggested that transfer planning would be easier if there was good transparency regarding the capacity utilization of lower care levels, a standardized transfer process, and improved interdisciplinary communication.

**Conclusion:**

To improve care transitions, transfer processes should be managed proactively across departments, and efforts should be made to identify and address care gaps.

## 1 Introduction

Discharge decisions for intensive care unit (ICU) patients are frequently taken under pressure to free up ICU beds. Without established guidelines or hospital Admission, Discharge, and Transfer (ADT) policies, evaluating the readiness to be discharged commonly relies on subjective judgments (1). In daily clinical practice, the challenge is to make the right decision at the right time for the right patient. A premature discharge to the ward can increase the risk for patients being readmitted to the ICU and may even elevate their risk of mortality (1). On the contrary, delayed discharge may waste resources and may result in the overtreatment of patients (2). In many countries, guidelines for patient discharge and transfers exist but are typically created locally. These guidelines often lack robust scientific foundations, fail to seamlessly integrate with hospital-wide patient flow procedures, and overlook relevant stakeholders' insights. Several studies have highlighted the need to define guidelines for ICU admission and discharge and to optimize the processes by involving the ICU and GW teams before and after discharge (3–5). Thus, structured insights into current clinical practices around patient transfers are needed to define the baseline and to implement any improvement measures. Therefore, the primary objective of this survey was to gain insights into the current status of ICU patient care transitions. We focused on transfer practices from the ICU, the involvement of stakeholders, transfer criteria, the established processes and tools used, the metrics related to ICU transfer processes, and current barriers to a timely and safe discharge. As a secondary objective, we intended to use the results to develop more specific guidelines for the standardization and optimization of care transition processes from the intensive care unit.

## 2 Methods

The study was conducted using a structured, web-based, anonymous, cross-sectional survey that is open to any participant from the target group, including practicing intensive care physicians, intensive care nurses, and bed coordinators in the acute care area. The participants' informed consent was obtained through dedicated questions before the start of the questionnaire. The survey was open for participation between 8 March 2022 and 19 September 2022. Participants could save their answered questions and continue later until the deadline of the survey. The survey was embedded in an online survey tool platform, adhering to the General Data Protection Regulation (welphi.com, Decision Eyes, Lisbon, Portugal). Ethical approval was obtained through a waiver by the Erasmus MC CRB (MEC-2022-0522). The survey was based on a systematic literature review (6) and the previous work of the research group (7–11).

The survey encompassed 29 questions, consisting of open, closed, and multiple-choice questions as well as five-point Likert scales (refer to Supplementary Datasheet 1). The questions were arranged in categorical blocks as follows: A. Demographics; B: Hospital size, type, and unit characteristics; C: Variables related to the current ICU discharge practice; D: Stakeholders and discharge decision-makers; E: Established discharge criteria; F: Discharge planning and discharge process; G: Occurrence of premature and delayed discharges; and H: Other reasons for suboptimal discharges that may relate to suboptimal care at the receiving unit, readmissions, or avoidable adverse events.

The survey was distributed in two languages: English and German. Both versions were pre-tested by eight test users per survey. Minor adjustments were made based on the feedback of pre-test users. No sample size calculation was performed, as the survey served as descriptive research. The survey was designed to maximize the completion rate and minimize dropout reasons. Therefore, only the minimum number of questions to generate the required insights were used. No iterative item reduction strategies were applied. The survey was endorsed by several intensive care societies [European Society of Intensive Care Medicine (ESICM), European Federation of Critical Care Nursing Associations (EfCCNa), Deutsche Gesellschaft für Fachkrankenpflege und Funktionsdienste e.V. (DGF), Deutsche interdisziplinäre Vereinigung für Intensiv- und Notfallmedizin (DIVI), and Deutsche Gesellschaft für Anästhesiologie und Intensivmedizin e. V. (DGAI)]. The link to the survey was distributed via the society's members lists. Data were summarized by a statistician using descriptive statistics. The absolute number and percentage of responses were calculated and used to interpret the opinion distribution for each question.

## 3 Results

### 3.1 Panel demographics

A total of 219 participants from 40 countries (with the majority from Germany, 48%; *n* = 105) participated in the survey held between March and September 2022 (see Supplementary Table 1). As questions were allowed to be omitted, completeness percentages of datasets ranged from 2 to 98% (with an average of 52% and a median of 45%), leading to variations in the total number of answers per question. An overview of the survey panel demographics and hospital and unit characteristics of the panelists' workplaces is provided in Table 1. Some outstanding specifics of the panel demographics were highlighted, with 63% of the participants being—wherever sex was specified—male individuals. ICU physicians were the largest professional group with 68% of the participants, followed by ICU nurses (24% of *n* = 200). The panel consisted of very senior ICU practitioners, with 147 specified leadership positions and long-term work experience in the ICU environment, with 40% having more than 20 years and 15% having 16–20 years of ICU experience.

**Table 1:** Survey panel demographics and hospital and unit characteristics.

**Survey panel demographics**					
Sex (*n* = 199 spec.)	Male 63%; *n* = 125	Female 37%; *n* = 74			
Professional group (*n* = 200 spec.)	ICU physicians 68%; *n* = 136	ICU nurses 24%; *n* = 48	ICU bed managers 1.5%; *n* = 3	Others 6.5%; *n* = 13	
Leadership positions (*n* = 147 spec.)	Senior physicians 48%; *n* = 71	Head of the department 38%; *n* = 56	Head of ICU nurses 14%; *n* = 20		
ICU work experience (*n* = 194 spec.)	>20 yrs. 41%; *n* = 7	16–20 yrs. 15%; *n* = 29	11–15 yrs. 20%; *n* = 38	5–10 yrs. 15%; *n* = 29	< 5 yrs. 10%; *n* = 19
**Hospital and unit characteristics of panelists' workplace**					
Hospital type (*n* = 193 spec.)	University hospital 38%; *n* = 74	Teaching hospital 30%; *n* = 58	Municipal hospital 14%; *n* = 26	Church hospital 9%; *n* = 17	Private hospital 7%; *n* = 13
Hospital size (*n* = 188 spec.)	>900 beds 25%; *n* = 47	600–900 beds 22%; *n* = 41	450–599 beds 15%; *n* = 29	250–449 beds 22%; *n* = 42	< 250 beds 15%; *n* = 29
ICU size (*n* = 176 spec.)	>25 beds 19%; *n* = 34	21–25 beds 11%; *n* = 19	13–20 beds 29%; *n* = 51	6–12 beds 40%; *n* = 70	< 6 beds 1%; *n* = 2
No. of patients treated in participant's ICU annually (*n* = 142 spec.)	>2,500 patients 11%; *n* = 16	1,001–2,500 patients 36%; *n* = 51	501–1,000 patients 27%; *n* = 38	251–500 patients 18%; *n* = 26	≤ 250 patients 8%; *n* = 11
Types of ICU (*n* = 185 spec.)	Interdisciplinary ICU 60%; *n* = 111	Surgical ICU 15%; *n* = 28	Medical ICU 11%; *n* = 19	Cardiological ICU 3%; *n* = 5	Neurosurgical ICU 3%; *n* = 5
The head of ICU (*n* = 160 spec.)	Intensivist 58%; *n* = 93	Anesthesiologist 30%; *n* = 48	Internist 4%; *n* = 7	Surgeon 2%; *n* = 3	Other 6%; *n* = 9

### 3.2 Hospital and unit characteristics

Two-thirds of the survey panelists worked in university and teaching hospitals. The largest group of panelists, constituting 25% of the total number of respondents (*n* = 188), worked in large hospitals with more than 900 beds. Small and medium-sized hospitals were equally represented. In total, 40% (of *n* = 176) of the participants worked in smaller ICUs with 6–12 beds and 29% worked in medium-sized ICUs with 13–20 beds. On average, 36% (of *n* = 142) of the panelists treated between 1,001 and 2,500 patients, and 27% of the panelists treated between 501 and 1,000 patients per year with their teams in their unit. The majority of the panelists worked in interdisciplinary ICUs (60% of 185). The units were mainly led by intensivists (58%) and anesthesiologists (30% of *n* = 160). When asked for other present units specialized in high-acute care in their hospitals, 153 panelists listed several other present units (*n* = 451 units listed), with the most commonly mentioned units being the intermediate care or step-down unit (24%) and the stroke unit (22%), followed by the palliative care unit (19%) and the chest pain unit (18%).

### 3.3 Performance metrics related to ICU discharge practice

The questions regarding routine ICU capacity utilization metrics revealed high ICU occupancy rates of ~90% (*n* = 69), with an ICU patient-to-nurse ratio of mainly 2 per day (44%) and 2–3 per night shift (49% of *n* = 160), an average ICU length of stay of ~4–5 days (41% of *n* = 69), and a readmission rate of ~5% (*n* = 46). Further metrics along the acute patient pathway were also high with a GW occupancy rate of ~90% (*n* = 38). In the GW, the patient-to-nurse ratios were observed to be 6–10 during the day (53% of *n* = 132) and 10–20 during the night shift (66% of *n* = 62). The average hospital length of stay was observed between 2.5 days and 10 days (67% of *n* = 52). About half of the participants that provided their ICU mortality stated it between 6% and 20% (51% of *n* = 49). Delirium rates were specified by 32 participants, where half of them reported delirium rates between 11 and 30% in their ICUs.

### 3.4 Stakeholders and discharge decision makers

Multidisciplinary rounds were used by the majority of the respondents (78% of *n* = 87). Reported participants were doctors, ICU nurses and physiotherapists. Only 33% (of *n* = 87) of the respondents consulted other departments to come to a discharge decision. In most cases, the other decision makers were the receiving unit and/or an infectious disease specialist. However, the final discharge decision was mainly taken by the ICU clinician on duty (43% of *n* = 190 votes; multiple answers possible; *n* = 91 panelists).

### 3.5 Established discharge criteria in daily clinical routine

Nearly half of the respondents (49% of *n* = 85) had ADT guidelines established in their hospitals, whereas 37% had none (14% did not know or could not answer). When asked for specific discharge criteria from the ICU to the GW or other lower levels of care established in their unit, the majority of the participants had none (56% of *n* = 85) and 36% had some in place (7% did not know). The respondents, who mentioned that specific discharge criteria were established, listed the type of patient-specific criteria that are usually considered for a discharge decision: patient's current acuity level, neurological status, and laboratory data were considered the most, followed by the patient's independence and mobility, frailty, and specific clinical scores [such as Early Warning Score (EWS), Modified Early Warning Score (MEWS), Acute Physiological and Chronic Health Evaluation Index II (APACHE II), Sequential Organ Failure Assessment (SOFA), Glasgow Coma Scale (GCS), Richmond Agitation-Sedation Scale (RASS), Confusion Assessment Method for the ICU (CAM-ICU), and Clinical Pulmonary Infection Score (CIPS), specified by *n* = 5].

Furthermore, the panelists were asked for the type of organizational criteria that were usually considered for a discharge decision. Current bed availability, patient-to-nurse ratio, competencies, and available technology at the receiving unit were mentioned the most, followed by the current acuity level in the ICU, available palliative care pathway outside the ICU, the current ICU occupancy rate, and the operating room (OR) schedule. Healthcare economic factors were strongly reported as not being considered when making discharge decisions. Of the respondents who had ICU discharge criteria in place, the majority (75% of *n* = 20) agreed that these criteria were specific enough to evaluate individual discharge readiness and ensure a safe transition to a lower level of care.

### 3.6 Discharge planning and discharge process

Panelists reported that most patients were discharged to a GW (73% of *n* = 84), whereas 23% were discharged to a step-down/intermediate care unit. A discharge protocol or handover form was used by 58% of the respondents (*n* = 49), mainly in a digital or a paper-based format (51 and 37%, respectively). A liaison nurse to facilitate the patient's discharge was reported by only 36% of the respondents (*n*=82). The majority of the respondents (63% of *n* = 82) reported the presence of a care gap between the time when a patient is no longer in need of ICU care and when a patient can safely transition to a GW. The proposed measures to decrease this care gap are shown in Supplementary Table 2.

Advanced discharge planning was deemed feasible by 80% (of *n* = 76) of the respondents, where there was no consensus on the optimal time window. The answers (*n* =28) were scattered between ≤ 6 h and up to >24– ≤ 48 h. Panelists who considered that discharge planning was feasible more in advance were currently lacking certain requirements that are listed in Supplementary Table 3.

### 3.7 The occurrence of premature and delayed discharges

The majority of the participants did not observe premature discharges from ICUs (57% of *n* = 83), while the remaining participants reported some premature discharges. The top three underlying reasons for premature discharges were ICU capacity strain, admission of patients with higher acuity, and lack of objective discharge criteria or scoring systems. On the contrary, delayed ICU discharges were common (68% of *n* = 82). The most cited reasons for delayed discharge were as follows: no free bed at the receiving unit; patient flow and discharge management processes not synchronized with the receiving units; lack of set care goals that need to be met for discharge readiness, and lack of specific discharge criteria (Supplementary Figure 1). Multimorbidity, delirium, and communication difficulties with the patient were mentioned as the most occurring reasons for the presence of suboptimal discharges that may relate to suboptimal care at the receiving unit, readmissions, or preventable adverse events (Supplementary Figure 2).

## 4 Discussion

The main goal of the survey study was to gain detailed and structured insights into the current process reality of ICU patient care transitions and its specific framework conditions. Based on the findings presented in this study, we believe that our research can enhance comprehension of the current baseline situation and play a role in formulating more precise guidelines to standardize and improve the transition of care processes from the intensive care unit. Addressing a pressing topic, especially in the past COVID-19 pandemic, the survey received great support after being endorsed and distributed by several intensive care societies. Additionally, the feedback received upfront on the survey content highlighted the need for more tangible insights into this area to establish standardized and cross-departmental acute patient flow management. This finding is supported by the related literature (5, 12–18), especially as study insights often result from rather small and regional to local cohorts. In many countries, the COVID-19 pandemic and retrospective analysis highlighted the present lack of clear discharge guidelines, specified admission criteria and acuity levels, and the match of acute care capacities with patient group needs (19–23). The survey was launched towards the end of the pandemic, and the ongoing appreciation for the topic was demonstrated by 219 participants from 40 countries, primarily holding senior positions and having long-term ICU work experience, who went through a list of 29 questions and provided quite a decent number of individual comments. In general, the participants' panel was dominated by ICU physicians with twice as many panelists as ICU nurses or other respondents. Academic hospitals represented most of the respondents' workplaces, and almost half of the panel worked in small units comprising 6–12 ICU beds. Interdisciplinary ICUs (60%) were the most common organizational form. ICUs were mainly led by intensivists or anesthesiologists. Intermediate care or step-down units, stroke units, and palliative care and chest pain units were common complementary units in the hospitals' high acuity area.

Based on the current literature, several performance metrics are associated with ICU discharge practice (1, 2, 24–30). Being asked about ICU performance metrics, acute care capacity strain with ICU and GW capacity rates of ~90% is a daily struggle, leading to not only premature discharges (pressure to free up ICU beds) but also delayed transfers (no available GW beds and no or too few IMC beds). In discharge decision making, multidisciplinary rounds within the ICU team have been established; however, the ICU clinician on duty takes the final decision in most cases. Only one-third of the participants practices shared decision-making across departmental borders (for example, with the receiving units) and consultation with other experts such as antibiotic stewardship members. Although being asked toward the end of the COVID-19 pandemic, still only half of the panel had ADT guidelines established in their hospitals, and only one-third of the panel had some specific ICU discharge criteria implemented. The latter consists mainly of the patient's individual vital parameters and laboratory data, patient status and autonomy, and institution-specific criteria, such as capacity metrics of the ICU and the receiving units, as well as available support resources at the lower care levels. Interestingly, the listed organizational discharge criteria match the top-perceived discharge barriers, leading to the assumption that positive patient flow drivers such as IMC and GW bed availability, specialized care capabilities in lower care levels, timely synchronized ADT processes, and OR schedules are considered for decision-making but are not yet implemented in the daily clinical routine. For patient transfer, mainly a discharge protocol or a handover form is used in a digital or a paper-based format. Liaison nurses are less common, especially in German-speaking countries but are top-listed as a measure to close the widely perceived care gap between ICU and GW care levels, next to establishing or increasing IMC capacities. Furthermore, 80% of the panelists believe that patient discharges can be turned from *ad hoc*-actions to in-advance planning and preparation, given good transparency of lower care level capacity utilization, predictability, and availability, as well as a standardized and implemented discharge planning process along with interdisciplinary communication. Suboptimal discharges may relate to suboptimal care at the receiving unit, negatively affecting the patient outcome and patient flow (5). Suboptimal discharges were mainly due to multimorbidity, delirium, and communication difficulties.

### 4.1 Limitations

As a large group of German respondents dominates the insights of this study, it would be very valuable to replicate the survey in several other local healthcare systems. Furthermore, the survey was dominated by ICU physicians and ICU nurses, and especially, bed coordinators were underrepresented. No GW staff was included, which could have provided further insights from the perspective of the patient receiver, for example, regarding the existing monitoring and care infrastructure, available skill sets, and patient–nurse ratios in the receiving units. With as many as 29 questions, this survey was rather extensive, thus resulting in small numbers of answers toward the end of the survey. Part B included hospital size, type, and unit characteristics, and part C included variables related to the current ICU discharge practice. We used open-answer types for text entry in both parts and asked the panelists to relate the provided data to a period. This strategy resulted in numerous incomplete answers that were difficult to analyze and statistically interpret. Furthermore, additional questions regarding the different patient pathways (emergency vs. non-emergency and medical vs. surgical) could have delivered insights into patient group-specific process differences, which might then support more targeted process optimization. In general, these study results can be used as a starting point to explore certain topics more deeply, perhaps with future dedicated mix-method studies.

### 4.2 Outlook

Given this kaleidoscope of insights into current ICU discharge practices, this study emphasizes the need for step-by-step implementation research. Although the drivers of positive patient flow are known and considered in decision-making, structured implementation measures to support safe and timely transfers are often not yet implemented. Cross-departmental and interdisciplinary efforts are still necessary to define common patient care goals along the patient pathway, communicate these goals effectively, monitor progress, and align them in context of organizational care capabilities. In addition, continuous organizational performance data provision is widely lacking to measure change. In a plan-do-study-act manner (31), long-term, step-by-step implementation research with a multistakeholder approach is needed to improve the overall patient flow. This might involve measures such as shifting capacities from ICUs to IMC or GW units, introducing new roles such as liaison nurses and central bed managers, implementing capacity utilization dashboards and clear discharge criteria checklists and handover forms, and aligning ADT planning and scheduling. Furthermore, the results could stimulate future research, for example, on collaboration and communication around care transitions within a hospital or even within regional care networks, discharge planning tools, process and performance benchmarking, and patient safety aspects associated with care transitions.

## 5 Conclusion

This survey study provided first insights into current ICU discharge practice on a broader multinational level. Critical issues, such as the pressure to free up intensive care beds and the lack of IMC and GW capacities, were listed by the majority of the participants. Furthermore, patients experienced rushed but also delayed discharges with all the negative side effects. For many study participants, established discharge criteria, cross-departmental decision-making, synchronized transfer processes, and adequate care capacity and quality at lower care levels were not the current reality in their daily clinical routine, although they were aware of its positive potential to improve care transitions. Here, good data on capacity utilization in acute medicine and insights into obstacles to patient flow can help to better control the use of existing capacities and align them with actual needs. To avoid bottlenecks in acute medicine and to improve patient flow, transfer processes should be managed proactively across departments based on structured and objective guidance. Gaps in care should be addressed by increasing IMC capacities and utilizing liaison nurses.

## Data availability statement

The raw data supporting the conclusions of this article will be made available by the authors, without undue reservation.

## Ethics statement

Ethical approval was obtained through a waiver by the Erasmus MC CRB (MEC-2022-0522), affiliated to the Erasmus Medical Center, being part of the Erasmus University Rotterdam. The studies were conducted in accordance with the local legislation and institutional requirements. The participants provided their written informed consent to participate in this study.

## Author contributions

MH: Conceptualization, Data curation, Formal analysis, Investigation, Methodology, Project administration, Validation, Visualization, Writing – original draft. CB: Data curation, Formal analysis, Methodology, Visualization, Writing – original draft. MW: Conceptualization, Formal Analysis, Investigation, Methodology, Supervision, Validation, Writing – original draft. HB: Conceptualization, Formal analysis, Investigation, Methodology, Supervision, Writing – review & editing. AK: Formal Analysis, Validation, Writing – review & editing. JB: Conceptualization, Formal analysis, Investigation, Methodology, Supervision, Validation, Writing – review & editing, Writing – original draft.
