# Supplementary material for: The current state of intensive care unit discharge practices - Results of an international survey study
Source: Front Med (Lausanne). 2024 May 7;11:1377902. doi: 10.3389/fmed.2024.1377902 (PMC11106471; doi:10.3389/fmed.2024.1377902)
Supplement: Supplementary file 1 [file Data_Sheet_1.PDF]

Supplementary Material

1 Document 1 online supplements: Survey in English language

Intro to the Welphi survey:

Thank you for taking the time to answer the following survey.

The aim of this survey is to gain insight into the current situation of ICU patient care transitions to the next lower level of care. The focus will be on transfer practices from the ICU, stakeholders involved, transfer criteria, established processes and tools used, metrics related to ICU transfer processes, and current barriers to timely and safe discharge to the next lower level of care.

With the results of this study, we want to create a basis for the development of more specific guidelines for the standardization and optimization of care transition processes from the intensive care unit.

The target group for this survey are intensive care physicians, intensive care nurses and bed coordinators in the acute care area.

Participation in this survey is voluntary and without any financial compensation. Opting out, but also a later completion of the survey is possible at any time. The survey results are evaluated completely anonymously.

Answering the survey takes about 15 minutes.

If you agree to participate, please click on "Next".

Informed consent:

I agree to participate in this survey.

I have understood that my participation is absolutely voluntary, I can opt out at any time and do not have to complete the survey.

I confirm that I have read the background information on this study and understood the aim of this questionnaire.

I understand that my data will only be used in anonymous form. My name will not appear in any report or publication related to this study.

If you agree with the above statements, please click on "I consent". If you do not agree, please click on "I do not consent".

Target audience for this questionnaire:

ICU physicians, ICU nurse, ICU bed manager,

A. Demographics of participants

1. Please indicate your profession:
☐ ICU physician
☐ ICU nurse
☐ ICU bed manager
☐ Other, please specify

2. Please indicate your gender.
☐ Female
☐ Male
☐ Prefer not to identify

3. Please indicate if any of the roles below apply to your current role?
☐ Head of Dept.
☐ Senior ICU physician
☐ Head of ICU nurses

4. How many years of work experience do you have in an ICU environment?
☐ < 5 years
☐ 5 – 10 years
☐ 11 – 15 years
☐ 16 – 20 years
☐ > 20 years

B. Hospital type, size and unit characteristics

5. In which type of hospital are you working?
☐ University hospital
☐ Teaching hospital
☐ Municipal hospital
☐ Private hospital
☐ Other, please specify:

6. In which country is your hospital located?

7. In which state is your hospital located?

8. How many beds does your hospital have?
☐ Up to 250
☐ 251 - 450
☐ 451 - 600
☐ 601 - 900
☐ > 900

9. What type of ICU are you working in?
☐ Surgical ICU
☐ Internal Medicine ICU
☐ Neurological ICU
☐ Neurosurgical ICU
☐ Cardiological ICU
☐ Interdisciplinary ICU
☐ Other, please specify:

10. How many beds does the ICU have, where you are currently working?
☐ < 6 beds
☐ 6 – 12 beds
☐ 13 to 20 beds
☐ 21 to 25 beds
☐ > 25 beds

11. How many ICU patients do you treat on average per year in your unit?

12. What is your average patient-nurse ratio in the ICU?

13. Who is leading your ICU?
☐ Anesthesiologist
☐ Intensivist
☐ Internist
☐ Surgeon
☐ Neurologist
☐ Other (please specify)

14. Please provide us with more insight in your high-acute care specialization by indicating which of the following units do you have in your hospital.
☐ CPU
☐ Stroke Unit
☐ Transplant-Unit
☐ Palliative Care Unit
☐ Intermediate Care Unit (IMC) or Step-down unit
☐ Other (please specify)

15. What is your average patient-nurse ratio at the general ward?

C. Variables related to current ICU discharge practice

Current literature indicates that the variables listed below have a relation with ICU discharge practice (1-9).

16. Please provide these numbers for your institution. Please specify from which time frame you have taken the numbers (e.g. average over past year, past two years, etc.).

  a. Average ICU-LOS in days
  b. Average hospital LOS in days
  c. Average ICU occupancy rate in % (please specify for your nominator, if you used listed beds or actually available beds e.g., not blocked because of isolation, staff or equipment shortages, etc.)
  d. Delirium rate (acquired during ICU stay) in %, if reported
  e. ICU mortality in %
  f. In-hospital mortality in %
  g. Readmission rate to ICU in % (if you have, pls. specify within 48 hrs. after discharge and in general)
  h. No. of cancelled surgeries because no ICU bed was available (pls. specify reporting period)
  i. Average general ward occupancy rate (please specify for your nominator, if you used listed beds or actually available beds e.g., not blocked because of isolation, staff or equipment shortages, etc.)

D. Stakeholders and discharge decision makers

17. In your unit, do you have multidisciplinary rounds (e.g. clinicians, nurses, physio-therapists, etc.) that consult on patient individual discharge readiness?
(yes / no)

18. In your unit, do you have functions from other departments (ethics representative, antibiotic stewardship member, infectiologist, representative from receiving unit) that consult on patient discharge readiness?
(yes / no)
(if yes, pls. name who is involved)

19. Who is finally taking the discharge decision?
(If the decision is taken by a group of people, please select all involved stakeholders)
☐ ICU clinician on duty
☐ Clinician on duty of the respective specialty
☐ ICU nurse
☐ Head nurse of the specialty / receiving ward
☐ Bed management
☐ Other, please specify

E. Established discharge criteria

20. Do you have Admission, Discharge and Triage-guidelines established in your hospital?
(yes / no / I don't know)

21. Do you have specific discharge criteria for discharges from the ICU to the general ward or other lower levels of care established in your unit?
(yes / no / I don't know)

a. If yes, what type of patient specific criteria are usually considered for a discharge decision?
☐ Vital parameter
☐ Lab data
☐ Patient's current acuity level
☐ Patient's diagnosis
☐ Patient's prognosis
☐ Patient's frailty
☐ Patient wishes
☐ Patient's independency and mobility
☐ Patient's neurological status
☐ Scores, if you use scores, please name the score(s):
☐ Further need for medication, therapy and monitoring
☐ Other, please name:

b. If yes, what type of organizational criteria are usually considered for a discharge decision?
☐ Current acuity level in the ICU
☐ Current occupancy rate in the ICU
☐ Current bed availability at the receiving unit
☐ Patient-nurse ratio, competencies and available technology at the receiving unit
☐ Palliative care pathway outside the ICU available
☐ OR schedule
☐ Healthcare economic factors
☐ Other, please name factor(s)

c. If yes, are these discharge criteria specific enough to evaluate patient individual discharge readiness and ensure a safe transition to a lower level of care?
Likert scale (very specific / specific enough / in parts specific and in parts vague / too vague for individual patient discharge readiness assessment / far too vague to guide discharge decisions / I can't judge)

d. If yes and you have voted in 21 c. that your current discharge criteria are not specific enough (or worse), what needs to be done / to be added to make them more specific?

F. Discharge planning and discharge process

22. To which next lower-level of care are your ICU patients generally discharged to?
☐ General ward
☐ Step-down unit / Intermediate care unit

 Other (please specify)

23. In your unit, is a discharge protocol or handover form being used?
(yes / no / don’t know)

a. If yes, which format does it have?
 Digital
 Paper-based
 Verbal checklist during rounds
 If other, please specify:

24. In your unit, do you have a liaison nurse in place that is there to facilitate the patient’s discharge and support the care team of the receiving unit?
(yes / no / don’t know)

25. In your institution, do you see a gap between the time when a patient is no longer in need for ICU care and when a patient can safely be cared for in a general ward?
(yes / no / can’t judge)

a. If yes, how could this gap be closed?

26. Do you think that planning the discharge of an ICU patient in advance is feasible in daily practice?
(yes / no / can’t judge)

a. If yes, what would be an appropriate time window between the start of planning a discharge and the actual patient transfer?
i. X hours

b. If yes, what are you currently missing to plan a patient’s discharge more in advance?

G. Occurrence of premature and delayed discharges

27. In your unit, do you believe there are patients being discharged prematurely?
(yes / no / I don’t know / can`t answer)

a. If yes, please name the reasons for premature discharges and rank them by their occurrence starting with the highest occurrence?

28. In your unit, do you have the feeling that patients’ discharges are delayed?
yes / no / I don’t know / can`t answer)

a. If yes, what are potential discharge barriers?
Multiple answers are possible.
(Answer possibilities: true / sometimes true / not true / I don’t know / can’t answer)
 Discharge decisions only taken during fixed time windows (eg. morning rounds)

☐ Lack of specific discharge criteria
☐ Lack of set care goals that need to be met for discharge readiness
☐ Key decision maker not available
☐ No free bed at the next lower level of care
☐ No contact person available at the next lower level of care
☐ Patient flow and discharge management not synchronized with processes at receiving units
☐ No transfer staff available
☐ No time for discharge, other ICU activities are prioritized
☐ Other (please specify)

H. Other reasons for suboptimal discharges (10) that may relate to suboptimal care at the receiving unit, readmissions or avoidable adverse events

29. If you have faced suboptimal discharges in your ICU that also relate to suboptimal care at the receiving unit, please select underlying reasons from the list below. Multiple answers are possible.
☐ Delirium
☐ Multi-morbidity
☐ Difficult to impossible communication with the patient
☐ No electronic patient file
☐ Practice variation due to the lack of specific ICU discharge criteria
☐ Lack of communication between ICU and general ward nurses
☐ Lack of communication between ICU and general ward doctors
☐ No coherent use of checklists
☐ Lack of awareness / focus / prioritization by management on well-organized /standardized / aligned patient transfers
☐ No culture of feedback between care teams / departments
☐ No or little structured consultation with the general ward
☐ Overestimation of the capabilities of the general ward
☐ Unavailability of the ward physician for face-to-face handover
☐ The ICU is perceived as an “island” within the wider hospital organization
☐ Lack of financial resources
☐ Lack of man hours/time
☐ Other (please specify)

Thank you page:

Thank you very much for your participation in this survey.

Maike Hiller

PhD student

Erasmus MC University Medical Center, Dept. of Intensive Care Adults, Rotterdam, The Netherlands

on behalf of the study group

References

1. Long EF, Mathews KS. The Boarding Patient: Effects of ICU and Hospital Occupancy Surges on Patient Flow. Prod Oper Manag. 2018;27(12):2122-43.

2. Badawi O, Breslow MJ. Readmissions and death after ICU discharge: development and validation of two predictive models. PLoS One. 2012;7(11):e48758.

3. <Badawi SCCM Late Breaker trimmed 02252018.pdf>.

4. Howell MD. Managing ICU throughput and understanding ICU census. Curr Opin Crit Care. 2011;17(6):626-33.

5. Hunter A, Johnson L, Coustasse A. Reduction of intensive care unit length of stay: The case of early mobilization. The Healthcare Manager. 2014;33(2):128-34.

6. van Sluisveld N, Hesselink G, van der Hoeven JG, Westert G, Wollersheim H, Zegers M. Improving clinical handover between intensive care unit and general ward professionals at intensive care unit discharge. Intensive Care Med. 2015;41(4):589-604.

7. Almoosa KF, Luther K, Resar R, Patel B. Applying the New Institute for Healthcare Improvement Inpatient Waste Tool to Identify "Waste" in the Intensive Care Unit. J Healthc Qual. 2016;38(5):e29-38.

8. Garland A, Connors AF, Jr. Optimal timing of transfer out of the intensive care unit. Am J Crit Care. 2013;22(5):390-7.

9. Bagshaw SM, Wang X, Zygun DA, Zuege D, Dodek P, Garland A, et al. Association between strained capacity and mortality among patients admitted to intensive care: A path-analysis modeling strategy. Journal of Critical Care. 2018;43(Feb. 2018):81 - 7.

10. van Sluisveld N, Oerlemans A, Westert G, van der Hoeven JG, Wollersheim H, Zegers M. Barriers and facilitators to improve safety and efficiency of the ICU discharge process: a mixed methods study. BMC Health Serv Res. 2017;17(1):251.
