# Supplementary material for: The current state of intensive care unit discharge practices - Results of an international survey study
Source: Front Med (Lausanne). 2024 May 7;11:1377902. doi: 10.3389/fmed.2024.1377902 (PMC11106471; doi:10.3389/fmed.2024.1377902)

Supplementary Material

2 Table 1 online supplements: Number of participants per country

**No. of participants per country** (n= 219 in total)			
**Europe**		**North & South America**	
Germany	(n=105)	USA	(n=3)
Netherlands	(n=7)	Brasil	(n=1)
Portugal	(n=6)	Colombia	(n=1)
Greece	(n=5)	Ecuador	(n=1)
Italy	(n=5)	**Middle East & Africa**	
Switzerland	(n=4)	Israel	(n=4)
Ireland	(n=4)	Saudi Arabia	(n=4)
Sweden	(n=3)	Bahrain	(n=1)
Denmark	(n=3)	Egypt	(n=1)
United Kingdom	(n=3)	Tanzania	(n=1)
France	(n=3)	Turkey	(n=1)
Belgium	(n=3)	**Central and East Asia**	
Spain	(n=2)	India	(n=6)
Bulgaria	(n=1)	Afghanistan	(n=1)
Croatia	(n=1)	Nepal	(n=1)
Estonia	(n=1)	Pakistan	(n=1)
Finland	(n=1)	Russia	(n=1)
Lithuania	(n=1)	Sri Lanka	(n=1)
Poland	(n=1)	**Australia**	(n=1)
Romania	(n=1)	**not specified**	(n=28)
Russia	(n=1)		
Serbia	(n=1)		
Slovenia	(n=1)		

3 Table 2 online supplements: Measures to close the care gap between ICU discharge and GW care level

Measures to close the care gap between ICU discharge and GW care level	n=44, multiple answers possible
Implement Intermediate Care Unit (IMC)	10
Liaison nurse	10
More IMC beds	8
More staff on GW unit	7
Faster / better organized care transitions / improved bed mgt.	6
Increase GW bed availability / Earlier discharge from GW to home	5
More GW beds	5
Improve care quality on GW	4
Handover checklist	3
Interdisciplinary rounding for a certain period post discharge at GW	2

4 Table 3 online supplements: Missing requirements for ICU discharge planning more in advance

Missing requirements for ICU discharge planning more in advance	n=21, multiple answers possible
Bed capacity at GW	5
Interdisciplinary communication to facilitate transfer	5
Advance discharge planning process implementation	4
Predictability	3
Transparency on bed availability at receiving units	2
Structured handover process with checklists, scoring, dedicated personnel	1
Clinical staff	1
Focus needs to change from focus on keeping patients longer in the ICU for reimbursement or bed reservation purposes	1

5 **Figure 1 online supplements: Discharge barriers that foster delayed ICU discharges**

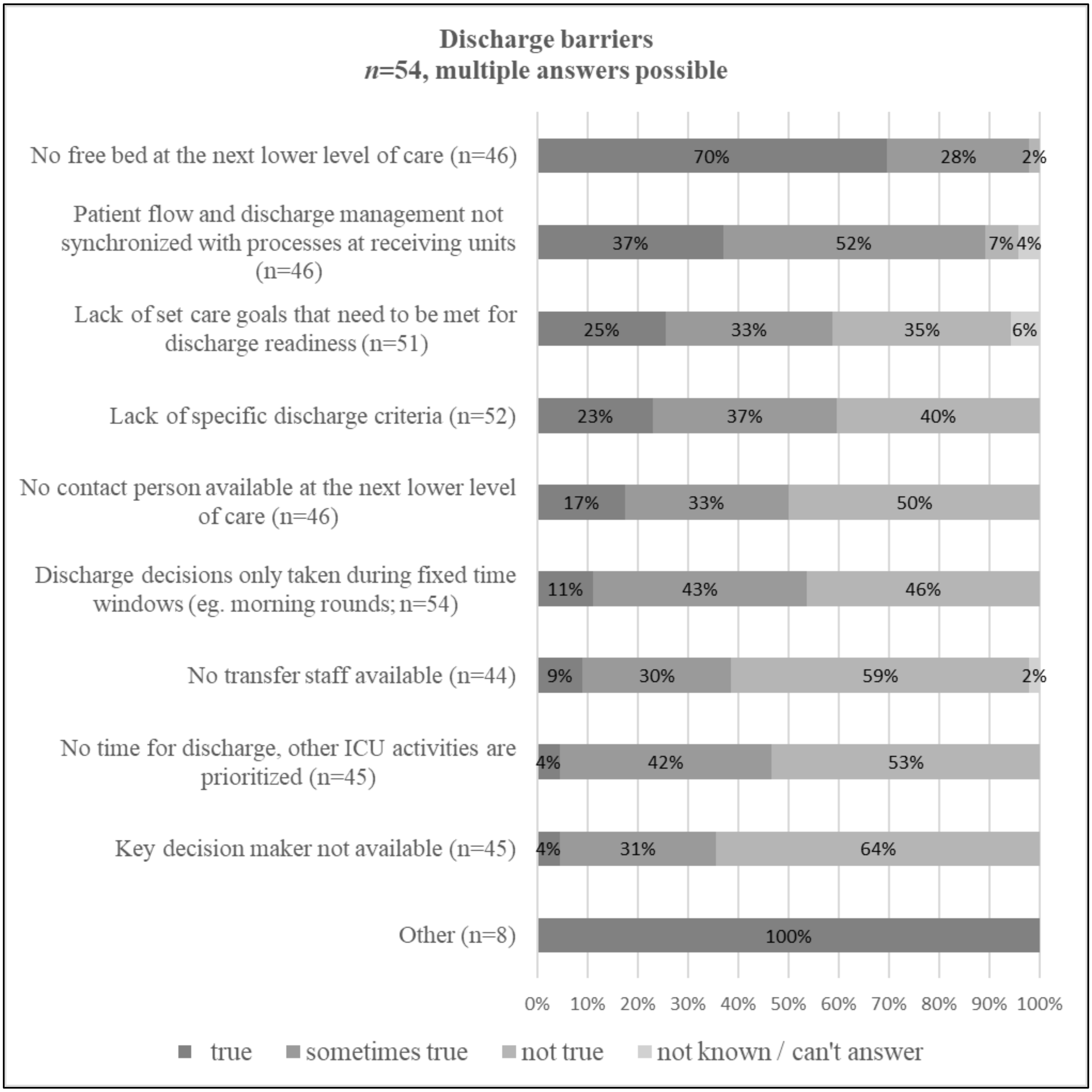

6 Figure 2 online supplements: Underlying reasons of suboptimal discharges

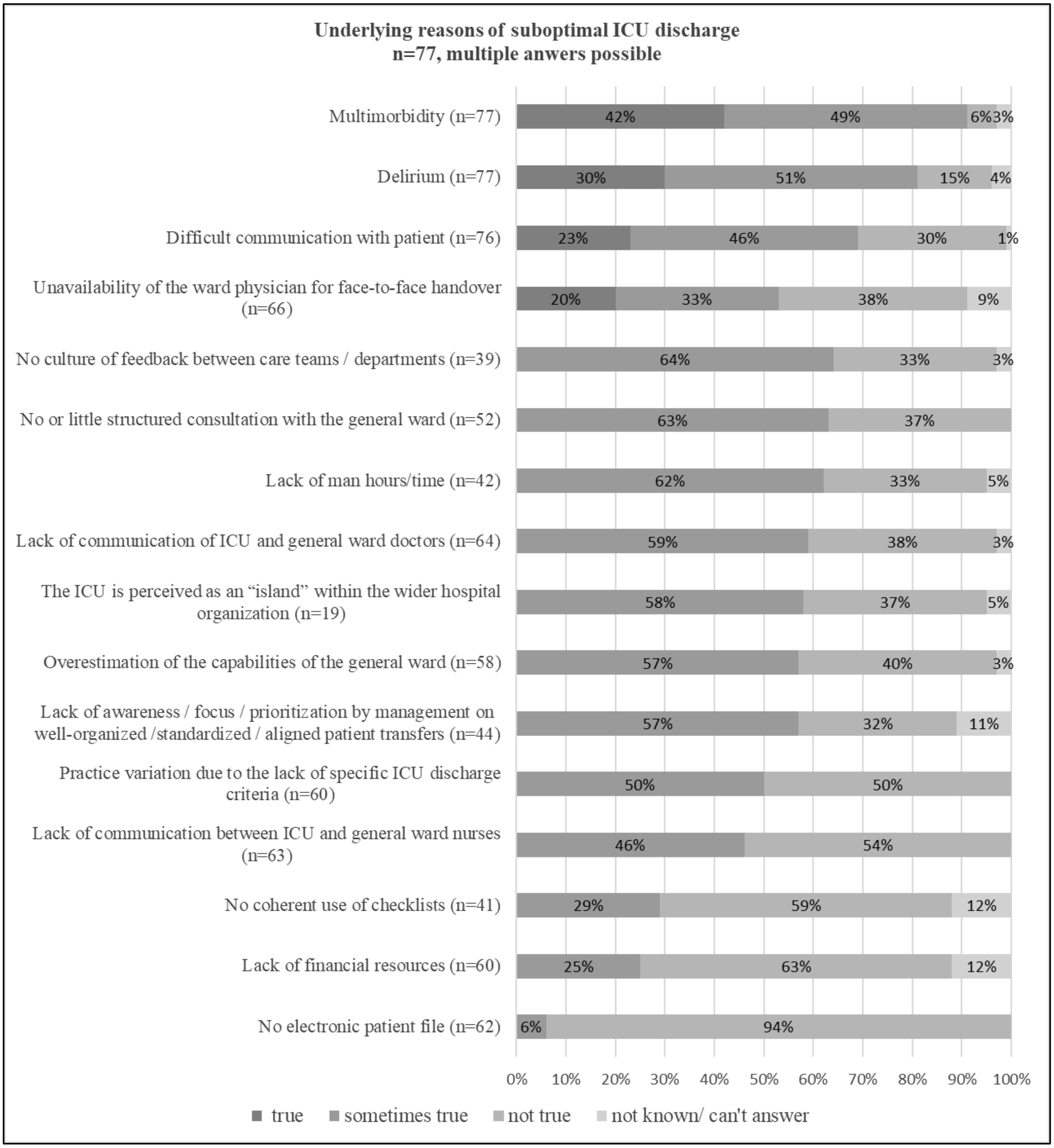

Supplement: Supplementary file 2 [file Data_Sheet_2.PDF]
